# PPE50 variants as novel phylogeographic signatures of host-pathogen co-evolution in tuberculosis

**DOI:** 10.1038/s42003-025-08383-3

**Published:** 2025-07-09

**Authors:** Christopher D’Souza, Jody E. Phelan, Paula-Josefina Gomez-Gonzalez, Joseph Thorpe, Taane G. Clark, Anthony G. Tsolaki

**Affiliations:** 1 Department of Biosciences, College of Health, Medicine and Life Sciences, Brunel University of London, London, UK; 2 London School of Hygiene and Tropical Medicine, London, UK

**Keywords:** Bacterial genes, Evolutionary genetics

## Abstract

While evidence supports co-evolution between *Mycobacterium tuberculosis* and humans, underlying mechanisms remain unclear. We identified PPE50 as a novel subfamily of PE/PPE proteins comprising eight variants. Surveying 387 *M. tuberculosis* complex (MTBC) strains representing global phylogeography, we found PPE50 variants are lineage-specific and stably associated with geographic regions, defining them as phylogeographically-associated proteins (PAPs). PPE50-381 is the ancestral variant (present in early-branching *M. canettii*) and the only variant observed in both Ancient and Modern MTBC lineages. Transcriptomic analysis confirmed that *ppe50* variant genes are expressed in strains from respective MTBC lineages, but not in all L1 strains and sub-lineages L2.1 and L4.1 where the gene was deleted. In silico analysis revealed significant structural diversity among variants, particularly in C-terminal regions. This strong association of *M. tuberculosis* protein diversity with phylogeography suggests PPE50 may contribute to MTBC adaptation to different host populations. Further characterization of PPE50 and other PAPs may facilitate improved targeted diagnostics, therapeutics and vaccines.

## Abstract (web summary)

PPE50 is a diverse novel subfamily of PE/PPE proteins in the *Mycobacterium tuberculosis* complex that show a phylogeographic distribution indicative host-pathogen co-evolution.

## Introduction

Tuberculosis (TB) remains one of the world’s deadliest infectious diseases, with an estimated 10 million cases and 1.3 million deaths annually^1^. TB is caused by the bacteria of the *Mycobacterium tuberculosis* complex (MTBC) which is comprised of *Mycobacterium tuberculosis* and *Mycobacterium africanum* that cause human disease, and others such as *Mycobacterium bovis* that cause disease predominantly in animals. In recent years, the explosion of genomic data generation has allowed us to map in detail the genetic diversity of the MTBC, identifying several distinct lineages for *M. tuberculosis* (L1-L4, L7-L10) and *M. africanum* (L5-L6), which are geographically stably associated with human host populations globally^2–7^. This phylogeographic pattern raises key questions about the mechanisms driving TB co-evolution and how specific pathogen proteins may facilitate adaptation to diverse human populations.

In this study, we investigated PPE50 (encoded by the *Rv3135* gene), a member of the PE/PPE protein family. This protein family is unique to mycobacteria and collectively represents approximately 10% of the *M. tuberculosis* genome coding capacity^8^. These proteins, characterized by N-terminal Pro-Glu (PE) or Pro-Pro-Glu (PPE) motifs, are often surface-exposed or secreted and show considerable sequence variation^9^. While their functions largely remain to be elucidated, evidence suggests roles in antigenic variation, host-pathogen interactions, and immune modulation^10^. Despite their potential importance, most studies have focused on reference strains (particularly H37Rv), potentially overlooking significant diversity across MTBC lineages.

PPE50 belongs to the PPE sub-lineage IV (SVP subfamily)^11^, and its genomic location is intriguing, lying in between the *Rv3134c* gene and *Rv3136* which encodes PPE51. *Rv3134c* is involved in the *dosR* regulon and biofilm formation^12,13^, whilst PPE51 has recently been described to form a heterodimer with the PE protein partner PE19, resulting in a cell surface complex involved in nutrient transport^14^. PPE50 has also been described as an immunogenic antigen in several studies, including during the chronic stages of infection in mice^15^, as a potent T-cell antigen in latent TB infection (LTBI) cohorts^16–18^, and as a potential vaccine candidate^19^. PPE50 has also been described as a potential drug target in several studies^20–22^. The potential immunological importance of PPE50 is further supported by studies showing that PPE50 can bind to Toll-like receptor 1 (TLR1) on THP-1 macrophages, upregulating anti-inflammatory responses via interleukin-10 (IL-10) induction^23^, and that PPE50 peptides are presented via major histocompatibility complex II (MHCII) by THP-1 macrophages infected with *M. bovis* BCG^17^. Moreover, PPE50/PPE51 peptides elicit significant interferon gamma (IFNγ) production in peripheral blood mononuclear cells (PBMCs) from both *M. tuberculosis*-infected individuals and patients with LTBI compared to those with active disease^18,19^.

Considering PPE50’s likely significance, we used a phylogeographic approach to determine the evolution of this protein in the MTBC to gain further insights into its function. Firstly, we investigated the genetic diversity of the *ppe50* locus (*Rv3134c-Rv3135-Rv3136*) in 18 reference strains from MTBC lineages^24^, and 6 animal-adapted MTBC strains. We identified significant genetic diversity in the *Rv3135* gene compared to neighbouring genes, resulting in eight distinct predicted PPE50 protein variants across the MTBC. Using a well characterised dataset comprising of 387 MTBC strains^5^, we further showed a stable lineage-specific association among PPE50 protein variants, thus defining them as phylogeographic-associated proteins (PAPs). Furthermore, we show that the *ppe50* variant genes are expressed in MTBC strains and through in silico analyses, have also predicted the 3D protein structural characteristics of the PPE50 variants to infer possible functions.

PAPs provide an approach for understanding how pathogens adapt to different host populations and are defined as proteins that show consistent sequence variations that correlate with pathogen phylogeny and geographical distribution, often reflecting adaptation to specific host populations or environments. In *Helicobacter pylori*, the CagA protein shows population-specific variations that affect host cell signalling and are associated with different gastric cancer rates across regions^25,26^. Similarly, the SasX protein is associated with specific Asian lineages of methicillin-resistant *Staphylococcus aureus* (MRSA) and contributes to colonization and virulence, demonstrating geographical clustering of protein variants^27^. In MTBC, proteins demonstrating PAP-like characteristics include PE_PGRS33, which exhibits sequence variations across lineage-specific clinical strains that elicit differential TLR2 responses, potentially altering host-pathogen interactions^28,29^. Additionally, the genetic diversity of the PPE38-PPE71 locus across MTBC lineages represents another important example^30,31^. Notably, PE_PGRS proteins require both the ESX-5 secretion system and functional PPE38 for secretion^32^. Several virulent *M. tuberculosis* strains, particularly from the L2 lineage, cannot secrete PE_PGRS and PPE-MPTR proteins due to loss-of-function mutations in the PPE38-PPE71 genetic locus^32,33^, presenting another potential PAP that may influence host-pathogen interactions in TB.

While other proteins show lineage associations, PPE50 represents, to the best of our knowledge, the first formally characterized PAP in the MTBC, introducing this concept to TB research. PPE50 forms a distinct protein subfamily with clear phylogeographic distribution, potentially playing a fundamental role in host-pathogen co-evolution. This variability across lineages, rather than a limitation, represents a potentially significant approach for therapeutic innovation. It may enable the development of region-specific interventions tailored to local MTBC populations, marking a paradigm shift from traditional approaches targeting only conserved antigens. Furthermore, PAPs may also facilitate the development of antigen ‘cocktail’ approaches that can target all strains of MTBC better than a single conserved antigen. PPE50 thus provides both a prototype for identifying other PAPs and a compelling foundation for developing lineage-specific vaccines and therapeutics that address the global geographical heterogeneity of TB, potentially enhancing diagnostics, prevention and treatment outcomes in specific populations.

## Results

### Structural variation at the *ppe50* (*Rv3134c-Rv3135-Rv3136*) genomic locus

**Fig. 1:**
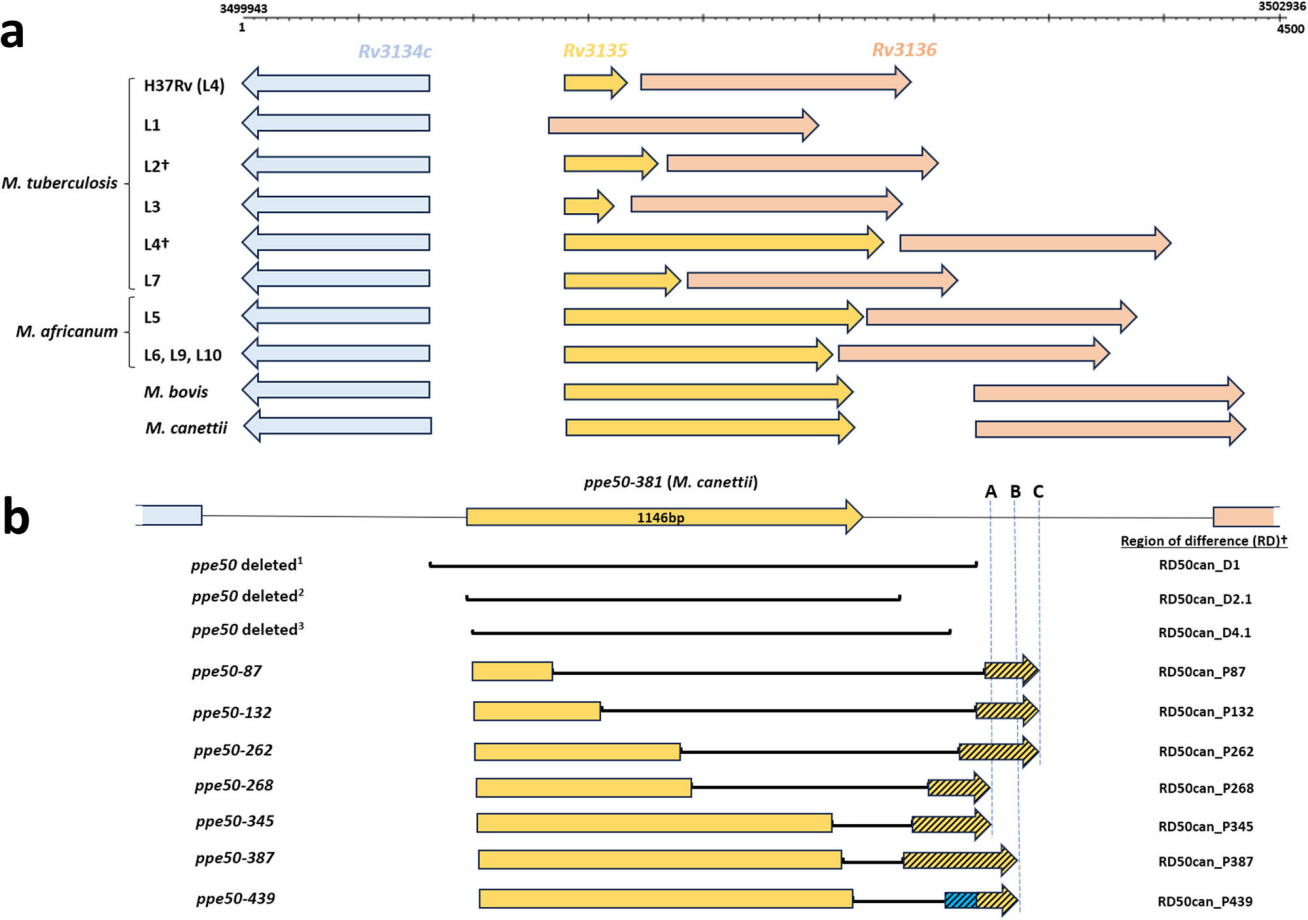
Genomic organization and structural diversity of *ppe50* variants in the MTBC. **a** Predicted open reading frames for *ppe50* variants and flanking genes. Genomic structural diversity in the *Rv3135* locus has resulted in several *ppe50* variant ORFs that are associated with MTBC lineages. *ppe50* gene length varies considerably in contrast to the *Rv3134c* and *Rv3136*, which are highly conserved. Genomic coordinates are shown with respect to *M. tuberculosis* H37Rv for ease of comparison. †: Note: *ppe50* variant ORFs for lineages L2.2.1 and L4.2.1.1. are shown only. **b** Regions of difference (RD) that form the *ppe50* variants in the MTBC. *ppe5*0 genomic locus for each variant were aligned to the *ppe50-381* locus of *M. canettii* (CIPT140010059). Deletions denoting RDs with respect to *M. canettii* are shown in black lines for each *ppe50* variant. In the *ppe50* variants, deletions span part of the *ppe50* gene and downstream intergenic region (hash arrows). ABC denotes base pair (bp) positions of the stop codons (downstream from the end of the *M. canettii* *ppe50-381* gene): (A) *ppe50-268* and *ppe50-345* (position 438); (B) *ppe50-387* and *ppe50-439* (position 520); (C) *ppe50-87*, *ppe50-132* and *ppe50-262* (position 590). *ppe50-439* has unknown 110 bp insertion (blue hash). *ppe50*-deleted 1, 2 and 3 are distinct  RDs for lineage 1, 4.1, and 4.2, respectively. **†**: Note, RDs described in this study and their coordinates are shown in the Supplementary Table 1.

An alignment of reference genomes covering MTBC lineages (L1-L10), *M. canettii* and other animal adapted strains (*M. bovis, M. microti, M. caprae* and *M. orygis*), revealed a remarkable contrast in diversity between the *Rv3135* (*ppe50*) gene and its flanking genes (Supplementary Fig. 1). Genes *Rv3134c* (dosR regulon) and *Rv3136* (*ppe51*) are highly conserved, with a small number of single nucleotide polymorphisms (SNPs) in their genes and intergenic regions. In contrast, there are large-scale insertions and deletions (indels) across the *Rv3135* gene, in reference strains, with respect to *M. tuberculosis* H37Rv. The *Rv3135* gene in all *M. canettii* and the animal MTBC strains are identical, except for a few SNPs, whilst in the *M. tuberculosis* reference strains, there is a range of diversity that appears lineage specific (Supplementary Fig. 1). In L1, *Rv3135* is completely deleted, whilst there are insertions, with respect to *M. tuberculosis* H37Rv, in L2, L5, L6 and *M. bovis*, which were also recently described^9^ (Supplementary Fig. 1). The variation in L3 was also previously described and was assigned to the principle genetic group 1 phylogenetic classification^34^. Recently, a comparative genomic study of MTBC strains also found regions of difference (RDs) that corresponded to L1, L2.1, L4.1 and L3 in the *Rv3135* genomic locus^35^. However, in the present study we have also identified RDs at the *Rv3135* locus that are linked with L7, L8 and L9 and several sub-lineages in L2 and L4 with respect to *M. tuberculosis* H37Rv (Supplementary Fig. 1). For each of the MTBC reference strains analyzed, we determined the open reading frames (ORFs) for this locus and found several genes for *Rv3135* (Fig. 1a). To better understand the evolution of this locus, all reference MTBC strains were aligned with respect to *M. canettii* and this revealed RDs with respect to *M. canettii* (RD50can), showing how the different variants of *ppe50* ORFs were formed (Fig. 1b). The extensive sequence diversity we have identified in *ppe50* variants has been previously unnoticed, as PPE50 (*Rv3135*) was primarily known only by the truncated 132-amino acid variant in *M. tuberculosis* H37Rv, which was presumed to be non-functional^36^. It is interesting to note that some *ppe50* variants have genes with identical stop codon positions (Fig. 1b). Furthermore, the deletions where *ppe50* is missing in lineages L1, L2.1 and L4.1 are all distinct in length, showing each to be a unique evolutionary event (Fig. 1b). Curiously, the 3’ end of the *ppe50-439* gene also has a 110 bp insertion (of unknown origin) that does not align to the intergenic region between *ppe50* and *ppe51* in *M. canettii* and is the only *ppe50* member to have this feature (Fig. 1b).

### PPE50 is defined by eight distinct variant proteins in the MTBC

**Fig. 2:**
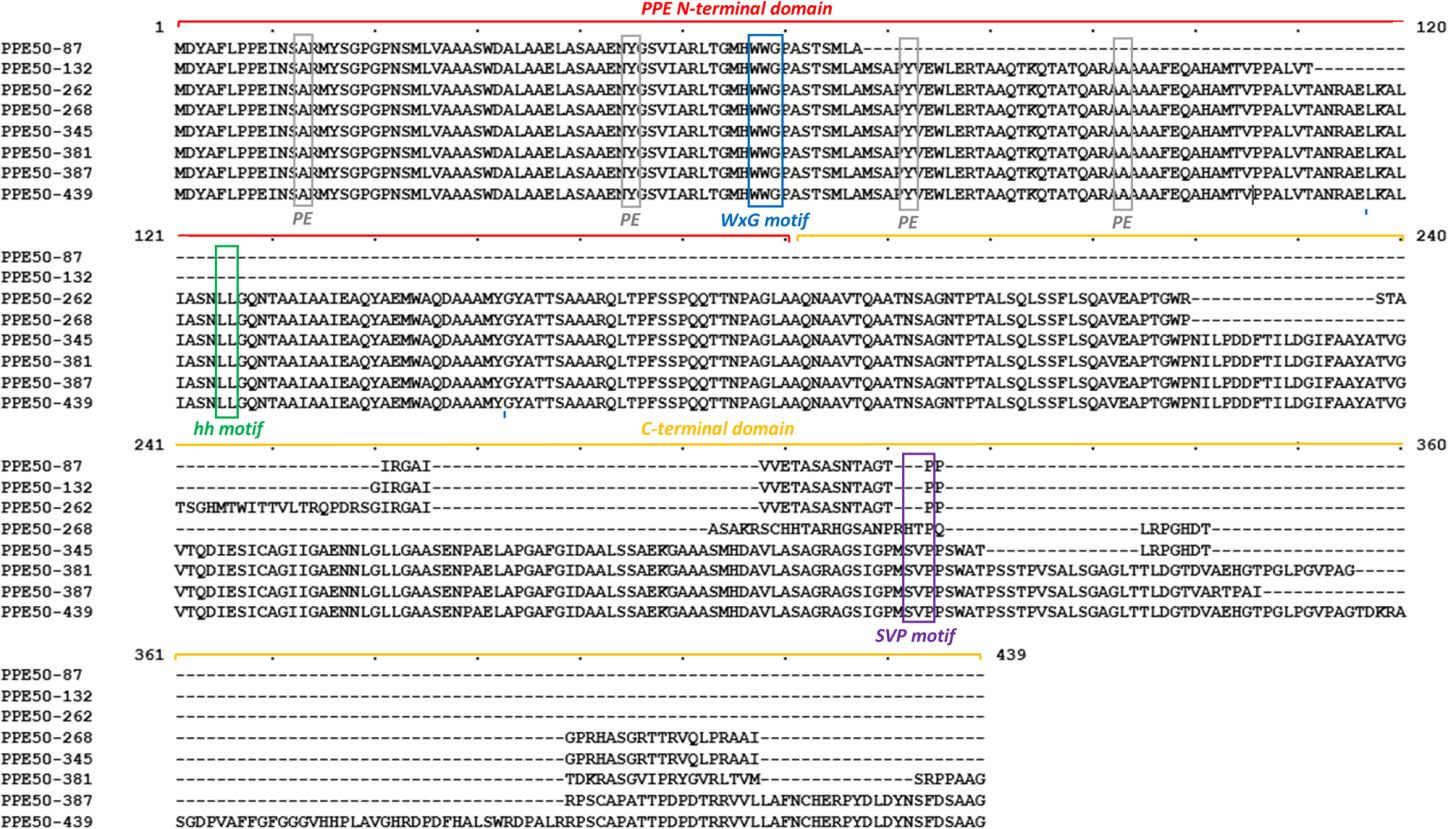
Multiple sequence alignment of PPE50 variant protein sequences. The predicted amino acid sequence for the eight PPE50 variants was aligned using MUSCLE. All variants have the WxG motif in their N-terminal domain. With the exception of PPE50-87 and PPE50-132, the remaining variants have the hh motif, a full-length PPE N-terminal domain and diverse C-terminal domains. PPE50-345, PPE50-381, PPE50-387 and PPE50-439 all have SVP motif in their C-terminal region. Residues involved in potential PE protein binding are shown in grey.

The genetic variation described in *Rv3135* has resulted in 8 predicted protein variants of PPE50, establishing a new subfamily for this PPE protein member. These protein variants range in size and molecular characteristics (Fig. 2). The shortest variant is PPE50-87, which is 87 amino acids long, followed by PPE50-132, which is 132 amino acids long. Both variants have all of their protein structure in the PPE N-terminal domain. The longest variant is PPE50-439 which is 439 amino acids in length and is comprised of the PPE N-terminal domain and a unique C terminal domain. Variants PPE50-262, PPE50-268, PPE50-345, PPE50-381 and PPE50-387 all have full length PPE N-terminal domains with unique C terminal domains that vary in length and sequence composition (Fig. 2). The size range of PPE50 protein variants is indicative of significant genetic variation, particularly in the C-terminal region. PPE50-87 and PPE50-132 are highly truncated variants, consisting of a partial PPE N-terminal region. Whereas PPE50-262 and PPE50-268 are semi-truncated variants, consisting of a full PPE N-terminal region and a relatively short C-terminal region. PPE50-345, PPE50-381, PPE50-387 and PPE50-439 are full length variants that are classified as PPE-SVP proteins, as they contain the serine-valine-proline (SVP) motif (residues 312-314) in their unique C-terminal domains (Fig. 2). These four additional PPE50-SVP variants increase the number of known PPE-SVP proteins, which are the largest subgroup of PPE proteins^11^. The presence of this motif is a key feature for substrates of the ESX-5 type VII secretion system^36^.

### Predicted structural diversity and sub-cellular location of PPE50 variant proteins

**Fig. 3:**
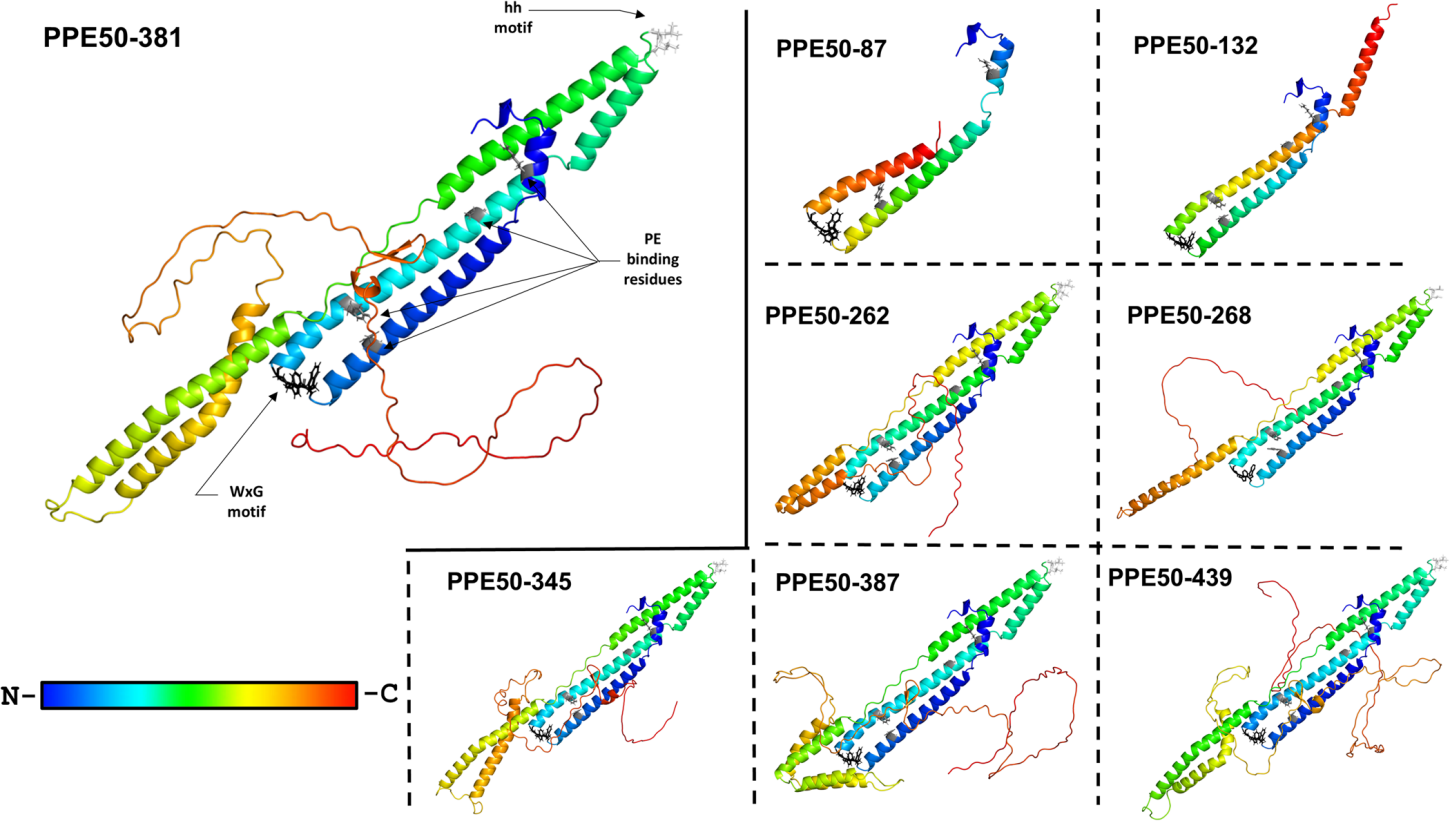
Predicted 3D structures of PPE50 variants. Ribbon structures were computed using AlphaFold. The N-terminal regions (partial or full-length) show the distinctive known alpha coils and turns of the conserved PPE N-terminal domain. With the exception PPE50-87 and PPE50-132, the disordered unique C-terminal domain is shown for the remaining PPE50 variants. Rainbow coloured gradient denotes N-terminal regions (blue) to C-terminal regions (red). Features are displayed with sticks: white = hh motif, black = WxG motif, and grey = PE binding residues.

Three-dimensional structures were predicted for the PPE50 variants (Fig. 3). All variants share the homologous PPE N-terminal domain structure, comprising of 180 amino acids forming 5 α-helices in a helical bundle-like conformation^37–40^. The PPE N-terminal domain structure also consists of several features. The WxG motif (residues 57-59 [WWG]) (Fig. 2), located between α-helices 2 and 3, likely forms a composite recognition structure with the PE C-terminal domain feature YXXXD/E, allowing for ESX type VII secretion ^41,42^. The hh motif or hydrophobic tip (residues 125-126 [LL]) (Fig. 2), is located between α-helices 4 and 5 (Fig. 3), and is essential for binding EspG, a cytosolic chaperone protein involved in the transportation of substrates via the ESX type VII secretion system^38,39,43^. All variants, except for truncated variants PPE50-87 and PPE50-132, have both the WxG and hh motif. Therefore, these variants are capable of forming, in part, the composite recognition structure needed to bind EspG, both of which are important for ESX type VII secretion over the inner mycobacterial membrane (Fig. 3). Whilst truncated variants PPE50-87 and PPE50-132 consist of only 3 or 4 α-helices, respectively, they do possess the WxG motif. However, it is unclear whether the WxG motif alone is sufficient for secretion.

Semi-truncated (PPE50-262 and PPE50-268) and full-length (PPE50-345 to PPE50-439) variants show unique C-terminal regions, differing in length, sequence composition, and consequently structure, as indicated by the TM-align scores (Supplementary Fig. 2). However, similarities can still be observed in the C-terminal regions of these variants, including a helix-turn-helix structure near α-helices 2 and 3 of the PPE N-terminal domain structure, except for PPE50-268, which has a single α-helix, and PPE50-387, which has two helix-turn-helix structures (Fig. 3). Another shared structure in the C-terminal regions of these variants, except for PPE50-262 and PPE50-268, is a two-stranded anti-parallel β-sheet near α-helix 2 of the PPE N-terminal domain structure. The C-terminal regions of all variants show a degree of disorder, compared to the ordered PPE N-terminal domain structure (Fig. 3).

Surprisingly, the C-terminal regions of these PPE50 variants also shroud the α-helices 2 and 3 of the PPE N-terminal domain structure, which are essential for binding with a PE family protein to form the canonical PE-PPE heterodimer (Fig. 3). As a result, this suggests that the semi-truncated and full-length PPE50 variants are unlikely to bind with a PE family protein, leaving PPE50-87 and PPE50-132 as the only variants with PE-PPE heterodimeric potential. However, it should be noted that the 3rd α-helix in PPE50-87 is significantly truncated, with only 2/4 PE binding residues (having residues 14[R] and 45[Y] and lacking 72[Y] and 93[A]), which may weaken or prevent dimerization^37^ (Fig. 2).

Hydrophobicity plots and transmembrane helix topology predictions were performed for the PPE50 variants (Supplementary Fig. 3). PPE50-87 was predicted to have only a cytoplasmic, transmembrane, and extracellular region, whilst all other variants were predicted to have a cytoplasmic, transmembrane, extracellular, and pore-lining helix region. The position of the transmembrane and pore-lining helix region is conserved between PPE50-262 and PPE50-268 (residues 118-133) and PPE50-345, PPE50-381, PPE50-387, and PPE50-439 (residues 237-252, except for PPE50-381, residues 238-253). Interestingly, the position of the N- and C-terminal regions varies between the cytoplasmic and extracellular regions, with PPE50-87, PPE50-345, PPE50-387 and PPE50-439 having a cytoplasmic N-terminal region and an extracellular C-terminal region, and PPE50-132, PPE50-262, PPE50-268 and PPE50-381 having an extracellular N-terminal region and a cytoplasmic C-terminal region.

### *ppe50* variants are strongly associated with distinct phylogeographic lineages of the MTBC

**Fig. 4:**
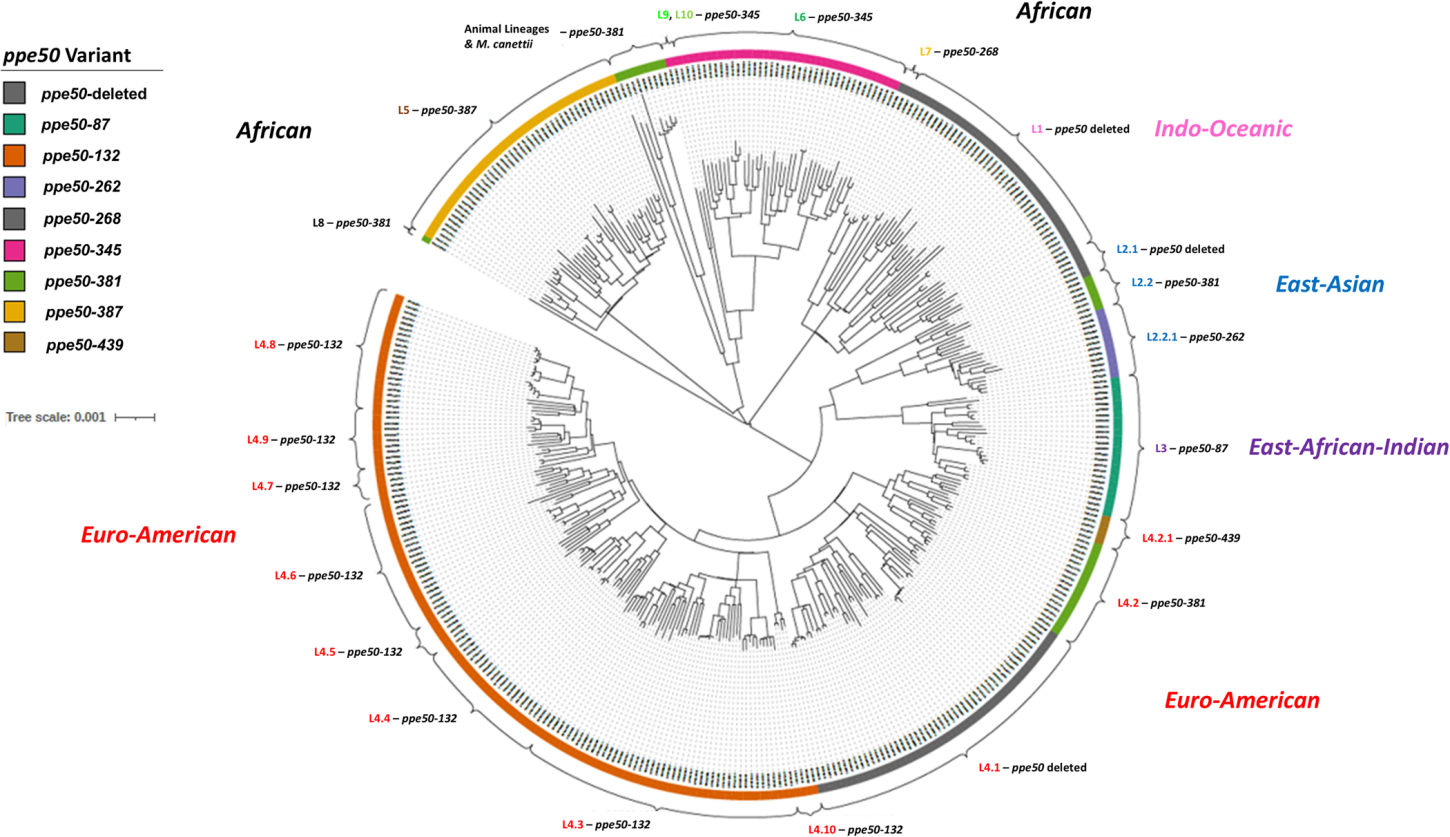
Phylogeograpic distribution of *ppe50* variants among 387 *M. tuberculosis* complex strains. Representative strains spanning MTBC lineages and sublineages were aligned (see Methods) and a tree produced using *FastTree2* software^96^ and viewed on iTOL (minimum of 5 strains per sub-lineage). Maximum Likelihood tree. Bar indicates genetic distance (nucleotide substitutions per site). The type of *ppe50* variant for each strain was identified by aligning the *Rv3134c*-*Rv3135*-*Rv3136* locus (using MUSCLE) and determining the *ppe50* ORF. The *ppe50* variant was then mapped to each MTBC lineage and sub-lineage. Therefore, this shows a stable phylogeographical association between MTBC lineage/sublineage and *ppe50* variant. Details of the MTBC strains studied are shown in Supplementary Data 1.

To investigate the phylogeographic distribution of *ppe50* variants in the MTBC, we analyzed an additional 387 well-characterized strains representing the global spread of the MTBC^5^. We identified the *ppe50* variant type for each strain and found a strong association between MTBC strain lineage, geographical location and *ppe50* variant (Fig. 4). Distinct predicted PPE50 variants proteins are found within distinct MTBC lineages and sub-lineages and have therefore been designated PAPs. To our knowledge, this is the first MTBC protein to be designated as a PAP. Although there is significant inter-lineage variation in *ppe50* variant genes, intra-lineage variation is minute showing high conservation of *ppe50* variants within their repective lineage/sub-lineage.

**Fig. 5:**
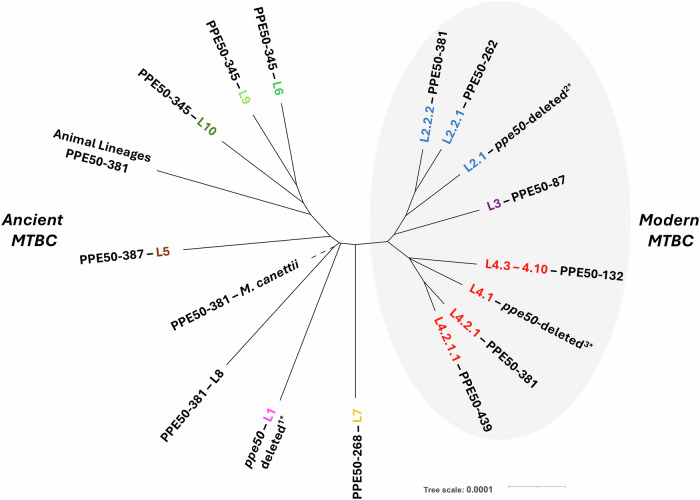
Evolution of PPE50 variants in the MTBC. PPE50 has evolved contrastingly between Ancient and Modern strains of the MTBC. PPE50-381 is the ancestral variant, being observed in *M. canettii*, and is the only PPE50 to be observed in Ancient and Modern MTBC strains. Recent expansion of Modern *M. tuberculosis* into lineages L2, L3 and L4 have seen marked diversity of PPE50 variants that are stably distributed in several sub-lineages that have spread globally. Whereas PPE50 variants in Ancient MTBC are mostly associated with Africa, except for L1. *Note: *ppe50* is deleted in lineages L1, L4.1, and L4.2 and each are unique event polymorphisms. Maximum Likelihood tree (implemented in IQ-TREE). Bar indicates genetic distance nucleotide substitutions per site for each MTBC lineage/sub-lineage.

Following the evolutionary history of the MTBC^44,45^, the distribution of PPE50 variants between Ancient and Modern MTBC strains is intriguing (Fig. 5). Only PPE50-381 is observed in both Modern and Ancient MTBC lineages, whilst the remaining PPE50 proteins are found exclusively in either Ancient or Modern MTBC (Fig. 5). Our data also shows that PPE50-381 is the ancestral protein of PPE50 subfamily, and is found in all *M. canettii* strains (an early branching tubercle bacilli)^46,47^. PPE50-381 is also found in animal-adapted MTBC strains and L8 strains (Fig. 5). *M. africanum* L5 and *M. tuberculosis* L7 strains have their own distinct PPE50 variants (PPE50-387, PPE50-268 respectively), whilst L6, L9 and L10 strains have PPE50-345. It is known that L5, L6, L7 and L9 strains are predominantly found in Africa and their PPE50 variants are all closely related to PPE50-381 (Fig. 5). Moreover, PPE50-268 and PPE50-345 have almost identical C-terminal domains, suggesting that they are evolutionarily related, except that PPE50-268 lacks the SVP motif (Fig. 2). In the Modern *M. tuberculosis* strains, denoted by the TbD1 deletion^44^, PPE50 variants show the most diversity (truncation), with expansion of several types in lineages L2 and L4 (Fig. 5). In contrast, PPE50-87 is the only PPE50 variant observed in lineage L3. In lineage L4, PPE50-132 is is found exclusively in several sub-lineages L4.3 to L4.10, which include *M. tuberculosis* strains that have expanded to North, Central and South America from Europe, e.g., the LAM clade^48^. PPE50-132 is also found in the reference strain *M. tuberculosis* H37Rv. Additionally in lineage L4, PPE50-439 is exclusively found in sub-lineage 4.2.1.1, whilst PPE50-381 is also observed in sub-lineage 4.2.1. PPE50-439 is the largest protein variant and it’s curious that is it observed only in a sub-lineage of the Modern L4 lineage, since the end of its C-terminal domain is identical to PPE50-387 which occurs in strains from the Ancient lineage 5 (Figs. 2 and 5). In lineage L2, PPE50-262 was exclusively present in sub-lineage 2.2.1, which includes the well-studied *M. tuberculosis* strain HN878, whilst PPE50-381 was also found in sub-lineage 2.2.2. As mentioned, in strains where *ppe50* is deleted (lineage L1, L2.1 and L4.1 strains), these deletions are distinct and have evolved separately. These include other well-studied strains of *M. tuberculosis*, e.g., CDC1551, Erdman and Haarlem (L4.1) which all have their *ppe50* genes deleted.

### *ppe50* variant genes are expressed in the MTBC

**Fig. 6:**
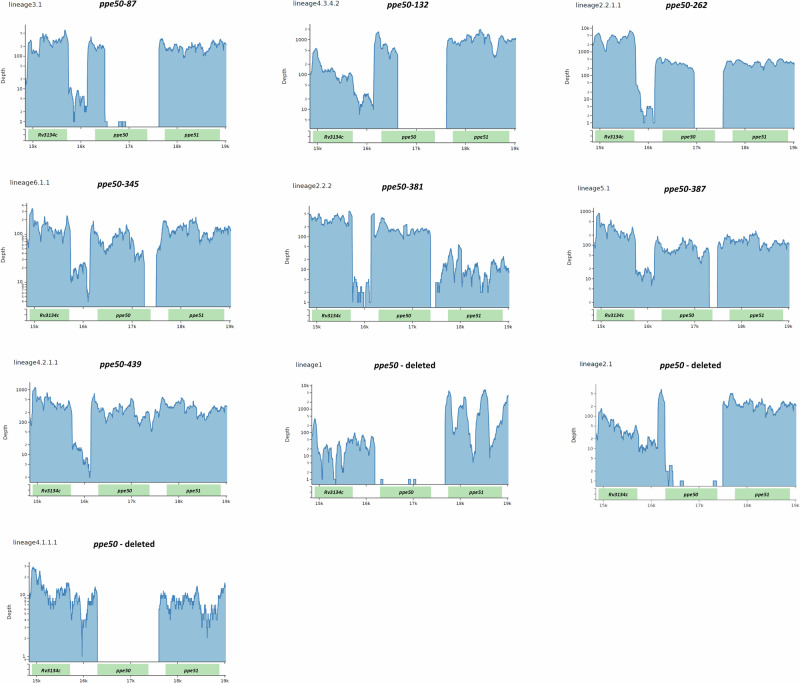
Transcriptome analysis of the *Rv3134c*-*Rv3135*-*Rv3136* locus showing the gene expression of *ppe50* variants. Transcriptome RNA-Seq reads from representative MTBC lineages for the *Rv3134c*, *Rv3135* (*ppe50*) and *Rv3136* (*ppe51*) genes were aligned to a reference locus containing the *ppe50-439* gene (lineage L4.2.1.1), since this is the longest *ppe50* gene. *ppe50* variant genes were shown to be expressed and this matched their sequence length. Transcriptome analysis not performed on *ppe50-268* from lineage L7. No expression was observed for all lineages where *ppe50* was deleted. Depth: Log scale of counts of transcript reads observed. x-axis scale: 1 kilobase increments for the locus.

Transcriptomic analysis of representative MTBC strains from each lineage revealed that the identified *ppe50* variant genes are expressed (Fig. 6; transcriptome analysis not performed on *ppe50*-268). Furthermore, the transcript length corroborates with the length of the *ppe50* ORFs that were predicted in this study. The depth of expression in each of the *ppe50* variants is comparable in intensity with the expression of the neighbouring genes *Rv3134c* and *Rv3136* (*ppe51*). Lineages where the *ppe50* gene was deleted showed no evidence of expression (Fig. 6). These data therefore suggest that these expression profiles are likely indicative of actual PPE50 proteins variants in MTBC that have been predicted and modelled in our study (Fig. 3 and Supplementary Fig. 3).

## Discussion

TB molecular epidemiology has revealed a distinction between globally distributed “Modern” *M. tuberculosis* strains and geographically constrained “Ancient” endemic strains. The TbD1 deletion, resulting in the loss of *MmpS6* and *MmpL6* genes, marks this evolutionary divergence and is present in all Modern lineages (L2, L3, L4) but absent in Ancient lineages (L1, L5-L10) and animal MTBC strains. This deletion appears to enhance bacterial fitness under oxidative stress and hypoxia, likely contributing to the successful global spread of Modern strains^44^. Similarly, we’ve identified PPE50 as a PAP that could also provide insights into TB’s global spread. These eight PPE50 variants show distinct MTBC lineage specificity, suggesting potential involvement in *M. tuberculosis* adaptation to specific host populations. While PPE50’s function remains unknown, our findings combined with experimental studies may reveal novel host-pathogen co-evolution insights. Notably, *ppe50* is deleted in certain lineages (all L1, L4.1, and L2.1). Clinical observations suggest contradictory outcomes: L1 strains are associated with increased pulmonary disease and cavitation^49^, yet *ppe50* deletion has also been linked to extrapulmonary TB^50,51^, and increased isoniazid and rifampicin tolerance^22^. This suggests *ppe50*'s loss may enhance virulence and drug resistance, analogous to the TbD1 deletion. Alternatively, *ppe50* may be essential only in specific MTBC genetic backgrounds, while lineages with *ppe50* deletions likely possess compensatory mechanisms, suggesting adaptive evolution rather than simple dispensability. Further experimental work is needed to test these hypotheses.

Nevertheless, the presence of PPE50 in its different variant forms may also give further insights into its biological role and interactions with the host. PPE50-381 is the ancestral variant protein of the PPE50 subfamily and is likely to have the most evolutionary conserved phenotype out of all the PPE50 variants, since it is the only PPE50 found in both Ancient and Modern MTBC strains. Indeed, PPE50-381 is also found in all divergent strains of *M. canettii* analysed in our study. Our in silico analysis suggests PPE50-381 is likely to be a cell wall protein capable of host interactions, supported by its presence in *M. bovis* BCG cell wall fractions^52^. A study reporting “*ppe50* gene replacement” in a meningeal-TB isolate^53^ actually described what our analysis identifies as a L2.2.2 strain naturally containing *ppe50-381*. Though this group episomally expressed PPE50-381 in *Mycobacterium smegmatis*, they observed no enhanced growth in THP-1 macrophages compared to vector controls^53^. However, it should also be noted that *M. smegmatis* lacks both PPE50 orthologs and the ESX-5 secretion system, potentially preventing proper PPE50-381 processing.

Most studies on PPE50 have been done on PPE50-132 (H37Rv strain) that is also found exclusively and at high frequency in lineages 4.3 to 4.10. This truncated PPE50, along with PPE50-87, could have a different biological role than PPE50-381 and other semi- and full-length PPE50 proteins, as they maybe more likely to form heterodimers with PE partners. The PPE50 C-terminal domains may shroud the crucial PPE α-helices 2 and 3 preventing PE interaction. Thus the loss of the C-terminal domain may act as “a molecular switch” promoting novel protein-protein binding and altered function. The PE-PPE heterodimeric conformation brings together the PPE WxG motif and the YxxxD/E motif of the PE C-terminal domain, likely forming a composite recognition structure for ESX type VII secretion^41,42^. Whilst this suggests that semi-truncated and full-length variants may not be secreted, their C-terminal regions are disordered, which may undergo conformational changes to enhance PE binding residue accessibility. Furthermore, other studies have reported type VII secretion system substrates being secreted without both WxG and YxxxD/E motifs and therefore, the three-dimensional structures of these motifs may be more important than the amino acids that define them^54^. Interestingly, the YxxxD/E has also been described as unstructured^37,43^. Therefore, semi-truncated and full-length PPE50 variants may not require a canonical PE partner, as the secretion signal may already be present within their disordered C-terminal regions, which also possess the flexibility to bring the motifs in close proximity, similar to the ESX-1 type VII secretion system substrate, EspB^55^. In either scenario, this would allow the composite recognition structure required for ESX type VII secretion to be formed. From the structural data in this study, all PPE50 variants are likely secreted over the inner mycobacterial membrane, except for PPE50-87 and PPE50-132, due to the absence of a hh motif. Moreover, PPE-SVP proteins are probable substrates of the ESX-5 type VII secretion system, suggesting PPE50 may also be secreted via this route^11,36^. The ESX type VII secretion system chaperone protein EspG also interacts with the hh motif, a feature that’s also present in all variants except PPE50-87 and PPE50-132^43^. EspG prevents PE-PPE heterodimer aggregation by binding and shielding the PPE hh motif, as well as stabilizing the complex in a secretion-competent state, but its essentiality has been challenged by the outer membrane/cell surface localization of a PE-PE (PE9/PE10) heterodimer lacking the hh motif^56^. PPE50 variants with SVP motifs may also be associated with sub-lineage IV ESX-5 secreted PE proteins, analogous to the recent findings for PE19/PPE51^14^. However, a recent study describes PPE50-132 from H37Rv being transcribed together with PPE51 and forming a proposed PPE-PPE heterodimer^23^ However, further data is needed given that PPE51 has also been reported to interact with PE19^14^. Our data suggest all PPE50 variants potentially cross the inner mycobacterial membrane, though the exact secretion mechanism requires further investigation. Structural analysis indicates these proteins likely localize to the cell surface, spanning from cytoplasmic to extracellular regions via a single transmembrane anchor.

Limited data on *ppe50* gene expression regulation (studied only in H37Rv) shows *Rv3135* is a commonly upregulated gene during environmental stress responses^57^. The gene shares putative transcription factor binding sites with *Rv3134c* and *Rv3136*^58^ and shows upregulation in *SigM* mutants^59^, suggesting involvement in cell surface and secreted molecule regulation during late infection stages. While *Rv3135* is not regulated by *DevR* under hypoxic stress^58^, a *PhoP* binding site exists in the intergenic region between *Rv3134c* and *Rv3135*^60^. Given *PhoP*’s role in intracellular survival and persistence^60,61^, *ppe50* expression may be influenced by host immune and antibiotic pressures, potentially resulting in variant-specific phenotypic responses to these stimuli.

PPE50 has also been identified as a potential drug target, with studies predicting interaction with the HIV-1 protease inhibitor Amprenavir^20^ and the compound GSK14022909A, which also targets proteins in the amino-acyl-tRNA biosynthesis pathway (*Rv1640c*, *Rv3598c*, *Rv3834c*) and peptide chain release factor 2 (*Rv3105c*)^21^. Additionally, a G251D mutation in *ppe50-381* from *M. bovis* BCG was associated with Florfenicol resistance, suggesting this residue’s functional importance^62^. These studies may provide further insights into PPE50 structure and function.

In this study, our phylogeographic approach revealed significant genetic variation in *Rv3135*, compared to the highly conserved *Rv3134c* and *Rv3136* flanking genes. These findings builds on previous observations showing limited *Rv3135* gene variation being associated with distinct principle genetic group 1^34^, and L1, L2.1 and L4.1^35^. Two other studies also identified *ppe50* variants in Beijing strains (L2)^63,64^, whilst a further study found PPE50 to be partially deleted in several *M. tuberculosis* isolates recovered from the Inuit^65^. Another study also found *ppe50* to be partially deleted in isolates belonging to L3, which were recovered from South Asians living in the United Kingdom^66^. The reasons for the genetic variation seen in *ppe50* are unknown. No evidence of homologous recombination or IS6110 involvement have been reported. The evolutionary timing of the divergence of *ppe50* across the MTBC is uncertain. While MTBC evolution estimates vary considerably (70,000-6000 years ago)^45,67^, archaeological evidence shows TbD1-containing *M. tuberculosis* strains existed in China and Europe 1000-2200 years ago^68,69^. This suggests *ppe50* divergence in Ancient MTBC likely predated the TbD1 deletion, while most *ppe50* diversification in Modern MTBC strains occurred subsequently during neolithic expansion from Africa^45^. After this, sub-lineages emerged, with MTBC strains L4.1 (*ppe50*-deleted^3^), L4.2 (PPE50-381, PPE50-439) and L4.3 to 4.8 (PPE50-132), calculated to have diverged in lineage L4 around the years 800 CE and 1000 CE and 1100 CE, respectively^69^. Similar findings were reported for L4.2, L4.4 and L4.5 in a recent study in China, in addition to L2.2 (PPE50-381, PPE50-262) which was found to have diverged around the year 806 CE^68^. Speculatively, these timelines suggest that the emergence of *ppe50*-deleted or PPE50-truncated types (PPE50-87, PPE50-132, PPE50-262) in MTBC strains may be a determining factor in the geographic spread of TB in densely populated areas driven by human migration over the last 2000 years (Modern L2, L3 and L4 MTBC). Whilst other longer PPE50 variants, (PPE50-345, PPE50-387), found in Ancient MTBC lineages L5, L6, L7, L9 and L10 represent the early evolution of PPE50 that may have contributed to the geographically restricted dissemination of TB in local host populations (e.g. Africa-India). However, the fact that PPE50-381 spans both evolutionary periods suggests that it may have contributed to the most highly adapted and versatile MTBC strains that are found in both animals and human populations that are the most globally distributed. Unlike the other MTBC lineages, lineage 4 strains have the widest global distribution and cause TB to a high frequency^70,71^. Sublineages 4.3 to 4.10 account for 71% of all L4 strains globally and all have PPE50-132, (with L4.3 accounting for 20.3%)^48^. Lineage L4.3 (also known as L4.3/LAM) originated in Europe and is the most widespread L4 sublineage spreading to Africa, Asia and the American continents^48^. The presence of PPE50-132 in a substantial proportion of L4 strains suggests that this PPE50 may be a key determinant of successful *M. tuberculosis* global spread. However, L4.5 and L4.6 (PPE50-132) have been reported to be more localised in their distribution (China and Central Africa, respectively)^48^. In contrast, sublineages L4.1 (ppe50-deleted^3^) and L4.2.1 (PPE50-381, PPE50-439) are present at lower frequencies globally, 19.1% and 4.4% respectively, with L4.1 also having a wide geographical distribution and L4.2.1 being confined to countries in Asia and Africa^48^. Taken together, it appears that truncation and/or loss of PPE50 in L4 strains maybe associated with increased global dissemination of those strains, although further sampling is needed to test this hypothesis. Lineage 3 *M. tuberculosis* strains (PPE50-87) originated in South Asia, but have spread elsewhere particularly to East Africa, probably as a result of human migration^72^. With the exception of Lineage 1 (*ppe50*-deleted^1^), all the Ancient MTBC lineage strains are geographically prevalent in Africa. MTBC lineage strains L6 (*M. africanum*), L10 and L9 are found on the Eastern side of West Africa, Central Africa and East Africa (Somalia) repectively and all have PPE50-345, showing a restricted spread^4,7^. MTBC lineage 5 (*M. africanum*) strains (PPE50-387) and lineage 7 strains (PPE50-268) are also geographically restricted, in Western Africa and Ethiopia respectively^4,73^, with unique PPE50 variants. *M. africanum* caused half of TB cases in West Africa, with older age, being HIV positive and malnurished as risk factors compared to *M. tuberculosis* L4 TB cases^74^. The distribution of full-length PPE50 variants, including PPE50-381 in MTBC lineage 8 from Rwanda^6^, appears to mirror the phylogeography previously described for Ancient MTBC. While this pattern suggests possible co-evolution with local African human populations, this apparent correlation may be coincidental and requires further investigation.

The evolution and phylogeographic distribution of PPE50 variants may be driven by host immunity, but T cell epitopes in MTBC are highly conserved^48,75,76^, suggesting PPE50 variation is unlikely to involve immune selection. Nevertheless, studies have demonstrated PPE50 immunological properties: PPE50-132 binds TLR1 on THP-1 macrophages, upregulating anti-inflammatory IL-10 responses^23^; PPE50-381 peptides are presented via MHCII in BCG-infected macrophages^17^; and PPE50/PPE51 peptides induce significant IFNγ production in PBMCs from LTBI individuals and TB patients^17,18^, with higher responses in LTBI. Even with these insights, the specific role of PPE50 variants on the immunopathology of TB  remains to be determined.

Despite the extensive genomic sequence data and structural variation documented for the MTBC, a critical challenge now is to determine the functional consequences of these genetic differences. Most studies involving PPE50 have focused solely on the variant found in *M. tuberculosis* H37Rv, which represents only one chapter in the broader PPE50 narrative. Future investigations must elucidate the phenotypic roles of different PPE50 variants and explain why this PPE protein subfamily exhibits such strong phylogeographic conservation. Our computational analyses, while robust, have inherent limitations. Future experimental work should test several key hypotheses, including whether C-terminal structural differences in PPE50 variants enable lineage-specific host protein interactions and if these variants trigger distinct immune responses, and how certain MTBC lineage strains function without PPE50. These studies would provide functional insights into these phylogeographic variations and potentially illuminate co-evolutionary dynamics between *M. tuberculosis* and human populations.

Identifying additional PAPs using our approach could reveal further candidates involved in host-pathogen co-evolution in TB. These PAPs represent promising preventative and therapeutic targets, as understanding their global distribution would clarify antigenic and metabolic variations across MTBC strains. Our findings emphasize the importance of studying diverse MTBC lineages rather than focusing solely on reference strains like H37Rv, CDC1551, and Erdman (which all lack PPE50). The discovery of lineage-specific PPE50 variants suggests opportunities for geographically targeted interventions that may overcome limitations of universal approaches like BCG in regions where specific lineages predominate. In conclusion, we’ve identified a subfamily of eight distinct PPE50 variants that are lineage-specific PAPs in the MTBC. While their precise function remains to be characterized, this work provides a template for discovering new PAPs in TB and other infectious diseases.

## Methods

### Reference genomes and datasets

The *ppe50* genomic locus (*Rv3134c-Rv3135-Rv3136*) was obtained from the *M. tuberculosis* H37Rv reference genome^8^. The 18 *M. tuberculosis* reference genomes (L1-L7) were obtained from a recent paper by Borrel et al.^24^. Reference genomes from *M. bovis* (AF2122/97), *M. bovis* BCG (1173P2), *M. microti* (OV254), *M. caprae* (spc-1), *M. orygis* (51145), and *M. canettii* (CIPT140010059 (STB-A)^77^, were obtained from the National Center for Biotechnology Information (NCBI) genomes database. Genomes from a subset of 387 MTBC strains were obtained from a recent paper^5^, where approximately 5 strains representing all known MTBC lineages and sub-lineages were selected for analysis. MTBC Lineages L8 and L10 reference genomes were also included^6,7^. Additionally, several more *M. canettii* strain genomes were also analyzed (CIPT140070010 (STB-K), CIPT140070017 (STB-J), CIPT140070008 (STB-L), CIPT140060008 (STB-D), ET-1291)^78,79^.

### Bioinformatic analysis

Genomic regions (*Rv3134c-Rv3135-Rv3136*) from reference genomes were aligned using Clustal Omega from EMBL-EBI^80^, and MUSCLE using MEGA X^81^. Alignments were visualized using the NCBI MSA Viewer 1.25.0. Phylogenetic reconstruction was performed using the Maximum Likelihood method within MEGA X^81^. Protein sequences were aligned using MUSCLE^82^ and visualized using MView from EMBL-EBI^83^. For large-scale phylogenetic analyses, representative strains for each sub-lineage were selected from the TB-Profiler webserver (tbdr.lshtm.ac.uk)^84^ (Supplementary Data 1). Raw fastq data for these samples was downloaded from the European Nucleotide Archive (ENA) site and mapped to the H37Rv reference genome using BWA (v0.7.17). Variant calling was performed for each sample individually using gatk HaplotypeCaller in gvcf mode (v4.1.4.1; parameters: -ERC GVCF). Join genotyping was then performed using gatk GenotypeGVCFs to create a single multi-sample vcf file. In-house Python scripts (https://github.com/LSHTMPathogenSeqLab/fastq2matrix/) were then used to transform this into a concatenated SNP fasta file which was used by IQ-TREE (v2.2.2.7; parameters: -m GTR + G + ASC) to perform phylogenetic reconstruction^85^. The phylogenetic tree was visualized using iTOL^86^. Assemblies were generated for each sample from raw reads using Shovill (v1.1.0) (https://github.com/tseemann/shovill), and PGAP (2023-10-03.build7061) was used to perform gene annotation^87^. A custom Python script was used to extract the *ppe50* gene from each of the MTBC strains using sequence similarity to the *M. tuberculosis* H37Rv *Rv3135* gene sequence, using BLAST, with a minimum match and identity threshold (https://github.com/jodyphelan/PPE50).

### Structural analysis

The tertiary structures of the PPE50 variants were predicted using Colabfold (Alphafold2 using MMseqs2)^88^ and the resulting PDB files were then visualized using ESPript V3.0 and PyMol 2.5^88,89^. Disordered regions and disordered binding regions were further defined using IUPred3 and ANCHOR2^90^.

### Functional and subcellular localization prediction

To predict the subcellular location of PPE50, hydrophobicity plots and transmembrane helix topology predictions were made using MEMSAT-SVM^91^. To predict the domains and function of PPE50, sequences were analyzed using MOTIF^92^, and MotifScan^93^ software tools. The presence of signal peptides was also predicted using SignalP ^94^.

### Gene expression analysis

To establish whether the different *ppe50* variant genes found across the sub-lineages are expressed, RNA-seq data in fastq format was downloaded from the ENA site and TB-Profiler and was used to infer sub-lineage for analyses^3,84^. Representative samples were selected and aligned to the genome assembly which contained the *ppe50-439* type (accession: ERR067597). Depth of coverage profiles were calculated with Samtools Depth (v1.12)^95^.

### Reporting summary

Further information on research design is available in the Nature Portfolio Reporting Summary linked to this article.

## Supplementary information

Supplemental Material

Description of Additional Supplementary Files

Supplementary Data 1

Reporting Summary

## Data Availability

All data supporting the findings of this study are available within the paper and its Supplementary Information. Genome Sequences of the MTBC strains analysed are provided in Supplementary Data 1.

## Code availability

A custom Python script was used to extract the *ppe50* gene from the MTBC strains. It is deposited at https://github.com/jodyphelan/PPE50.
