# Supplementary material for: PPE50 variants as novel phylogeographic signatures of host-pathogen co-evolution in tuberculosis
Source: Commun Biol. 2025 Jul 9;8:1024. doi: 10.1038/s42003-025-08383-3 (PMC12241500; doi:10.1038/s42003-025-08383-3)
Supplement: Supplementary file 1 — Supplemental Material [file 42003_2025_8383_MOESM1_ESM.pdf]

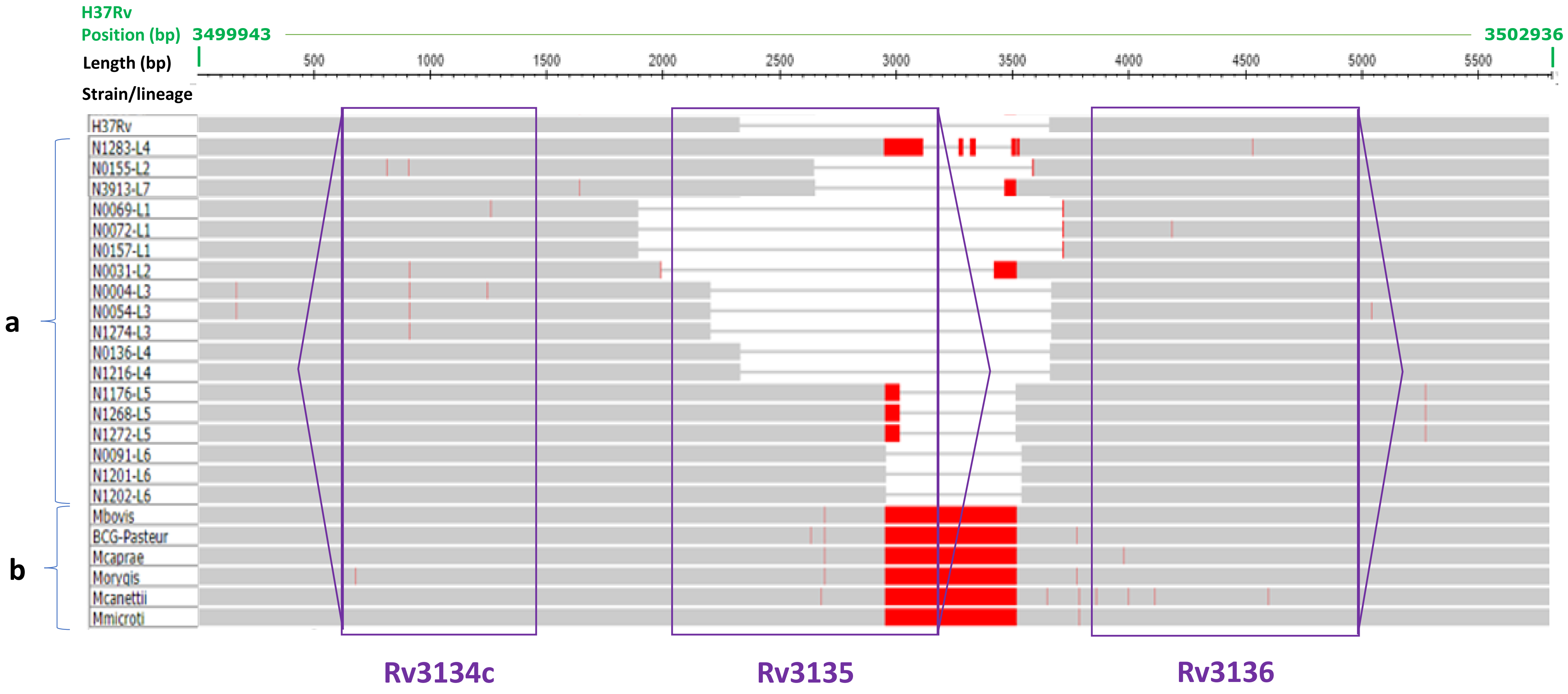

Supplementary Figure 1: Structural diversity at *Rv3134c-Rv3135-Rv3136* locus. Regional alignment of locus using MUSCLE with respect to *M. tuberculosis* H37Rv and viewed on NCBI MSA viewer. **a**. MTBC lineage reference strains from *Borrel et al.,* [24]. **b.** Representative animal MTBC strains. Red annotations show sequence differences, SNPs and insertions/deletions across the locus. Both *Rv3134c* genes and *Rv3136* are highly conserved across the difference lineages and animal strains. However, *Rv3135* is highly variable with the pattern of diversity being lineage specific. Note the position of the *Rv3135* ORF is approximate for all strains except *M. tuberculosis* H37Rv.

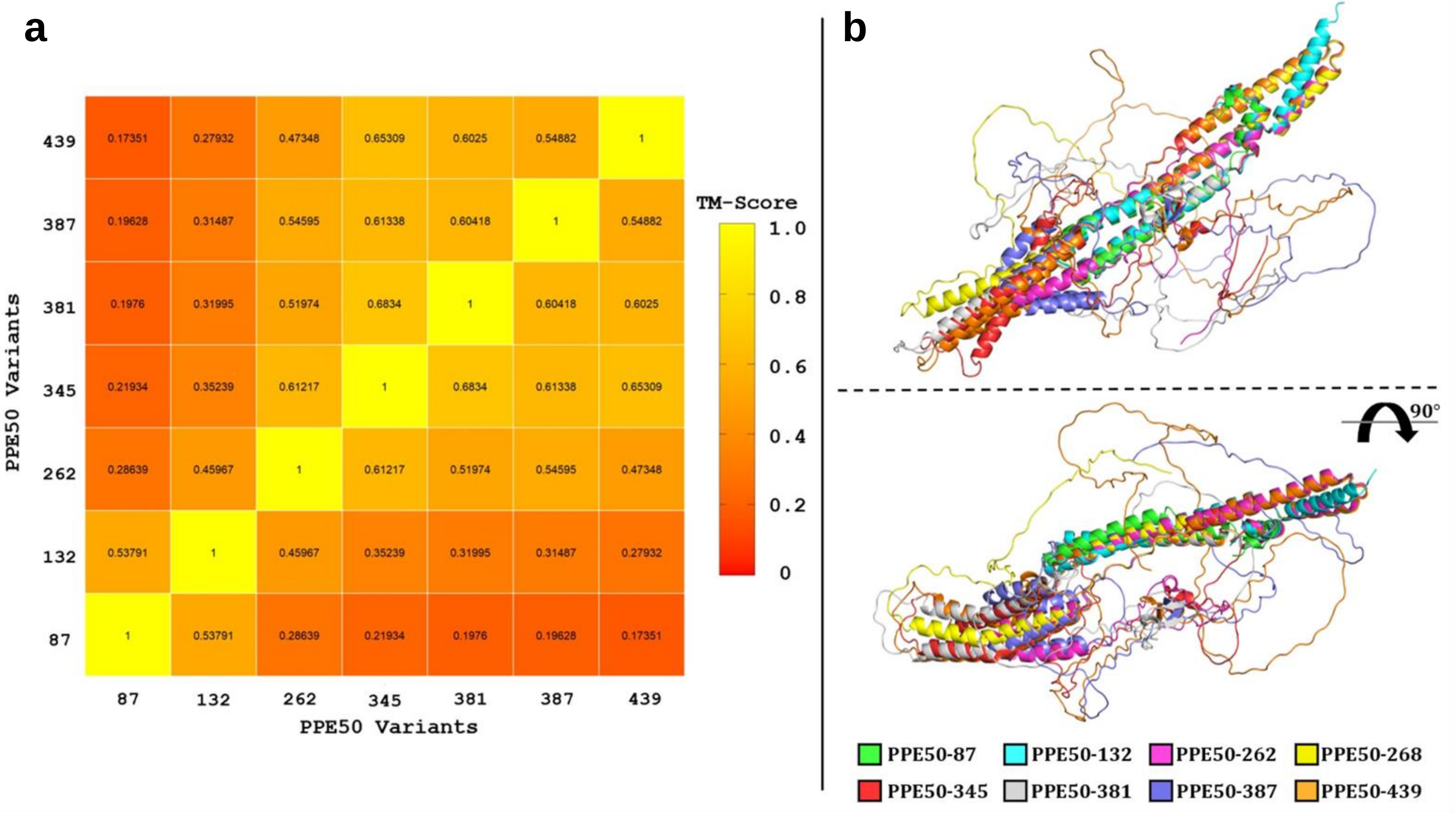

Supplementary Figure 2: a. TM-align highlight structural diversity. **b.** Side and top view images of all PPE50 variant structures superimposed. Colours: PPE50-87 = green; PPE50-132 = cyan; PPE50-262 = pink; PPE50-268 = yellow; PPE50-345 = red; PPE50-381 = white; PPE50-387 = violet; and PPE50-439 = orange. All structures were aligned against H37Rv (PPE50-132).

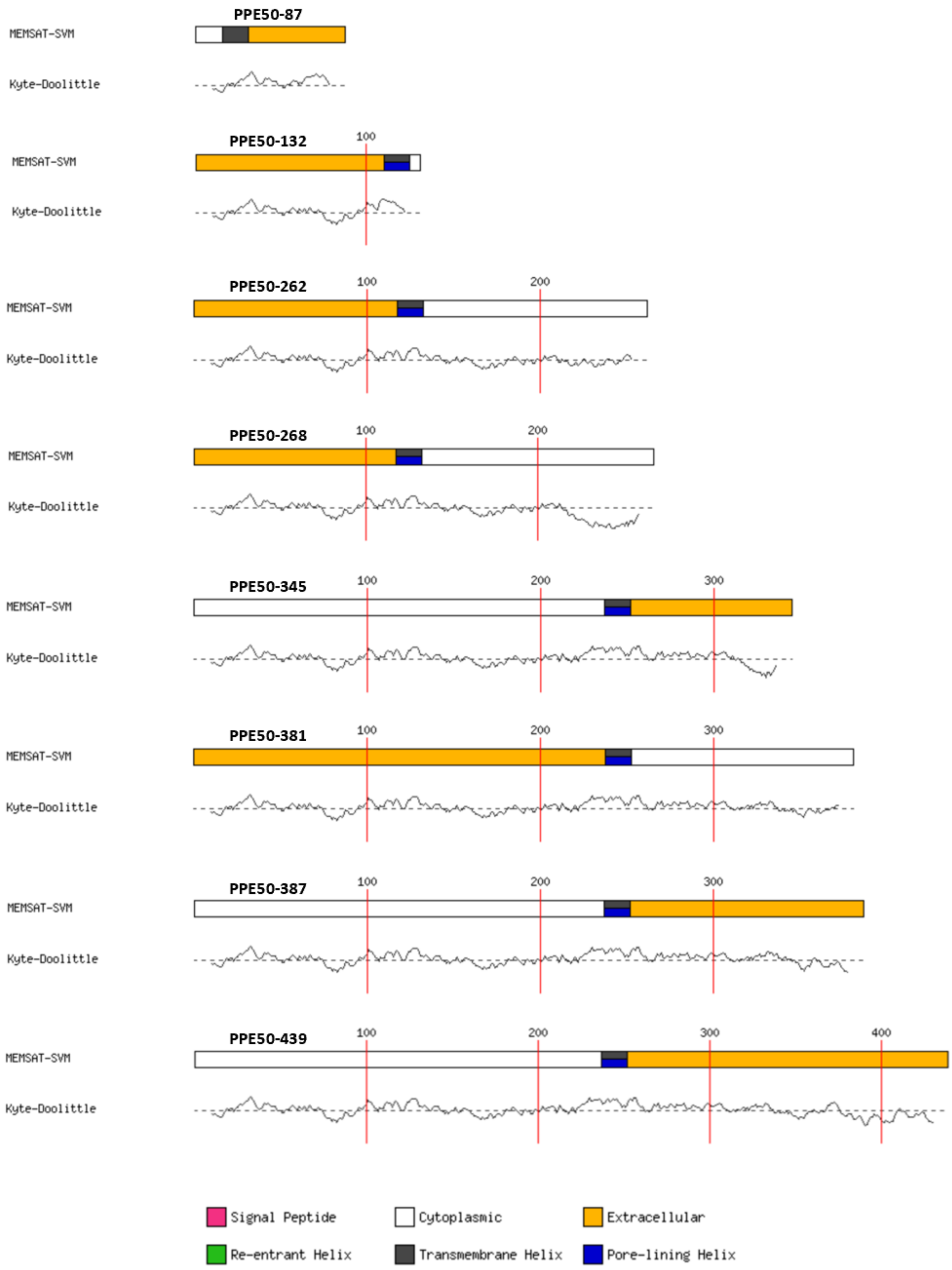

Supplementary Figure 3: Hydrophobicity plots and transmembrane helix topology predictions for PPE50 variant proteins. Schematics encompassing both hydrophobicity plots and transmembrane helix topology predictions. The Kyte-Doolittle hydrophobicity plots were produced using ProtScale (hydrophobic regions (above the dotted line) or hydrophilic regions (below the dotted line). The transmembrane helix topology predictions and schematics were then produced using MEMSAT-SVM. This software is also able to predict extracellular or cytoplasmic regions as well as signal peptides.

Supplementary Table 1: Deletion characteristics that form the PPE50 variants genes in the MTBC

PPE50 variant	Region of Difference (RD)	Representative lineage	Deletion Start	Deletion Stop	Stop codon of PPE50 variant gene
PPE50-deleted[1]	RD50can_D1	L1	-119	+1728	-
PPE50-deleted[2]	RD50can_D2.1	L4.1	+1	+1650	-
PPE50-deleted[3]	RD50can_D4.1	L2.1	-9	+1429	-
PPE50-87	RD50can_P87	L3	+200	+1672	+1736
PPE50-132	RD50can_P132	L4.3 (H37Rv)-4.10	+331	+1668	+1736
PPE50-262	RD50can_P262	L2.2.1	+653	+1600	+1736
PPE50-268	RD50can_P268	L7	+657	+1479	+1629
PPE50-345	RD50can_P345	L6, L9, L10	+956	+1547	+1629
PPE50-387	RD50can_P387	L5	+1017	+1519	+1666
PPE50-439	RD50can_P439	L4.2.1.1	+1084	+1210 + insertion[†]	+1666

Note: Coordinates are relative to start codon of *M. canettii* PPE50-381 gene - 1146bp (see Figure 1b)

[†]: 110bp insertion at position 1210 to 1540 (unknown sequence)
