# Supplementary material for: PPE50 variants as novel phylogeographic signatures of host-pathogen co-evolution in tuberculosis
Source: Commun Biol. 2025 Jul 9;8:1024. doi: 10.1038/s42003-025-08383-3 (PMC12241500; doi:10.1038/s42003-025-08383-3)
Supplement: Supplementary file 2 — Description of Additional Supplementary Files [file 42003_2025_8383_MOESM2_ESM.pdf]

# Description of Additional Supplementary Files

**File name:** Supplementary Data 1

**Description:** Characteristics of MTBC strains used in this study
